# Oral lichen planus: case series and experience in a tertiary dermatology service in Brazil^☆^

**DOI:** 10.1016/j.abd.2022.06.005

**Published:** 2023-03-22

**Authors:** Aline Erthal, Silvia Vanessa Lourenço, Marcello Menta Simonsen Nico

**Affiliations:** a Department of Dermatology, Faculty of Medicine, Universidade de São Paulo, São Paulo, SP, Brazil; b Department of Pathology, Faculty of Dentistry, Universidade de São Paulo, São Paulo, SP, Brazil

**Keywords:** Lichen planus, Oral lichen planus, Oral mucosa

## Abstract

**Background:**

Lichen planus is an inflammatory disease that can affect both the skin and mucous membranes, including the oral mucosa. There is very little original Brazilian dermatology literature about oral lichen planus.

**Objective:**

To describe the clinical, pathological, and treatment data of 201 patients diagnosed with oral lichen planus followed at the Stomatology Outpatient Clinic of Hospital das Clínicas, Universidade de São Paulo, from 2003 to 2021.

**Method:**

The patients demographic profile, the morpho-topographic features of the lesions, the treatment employed, and the possible presence of squamous cell carcinoma were analyzed.

**Results:**

The disease was more common in women over 50 years of age, tending to be chronic, with a large number of cases showing cicatricial sequelae in the mucosa. Topical treatment with potent corticosteroids was shown to be effective in the vast majority of cases. Squamous cell carcinoma in oral lichen planus cicatricial sequelae was observed in eight cases.

**Study limitations:**

Retrospective study of medical records, with gaps regarding the filling out of data; unequal observation time among the studied cases.

**Conclusions:**

This is the largest Brazilian dermatology series on oral lichen planus. The response to topical corticoid therapy was excellent in the vast majority of cases. The high prevalence of atrophic lesions, demonstrating the chronicity and tissue destruction potential of this disease, may explain the large number of cases of squamous cell carcinoma.

## Introduction

Lichen planus is a frequent inflammatory disease of unknown cause, which can affect both the skin and the mucous membranes, including the oral mucosa. Oral lichen planus is believed to affect 0.5% to 2% of the adult population, with most of them being mild cases^1^. It is known to occur more frequently in women and affects all ethnicities, although it has been more frequently observed in Caucasians in the very few series in which the latter aspect was documented^2^. While lichen planus skin lesions are generally self-limited, almost always occurring in outbreaks, oral lichen planus (OLP) lesions more commonly tend to be chronic and persistent if untreated; thus, they may develop cicatricial sequelae, similar to what occurs with other protracted forms of the disease, such as ungual LP (anonychia) and scalp LP (alopecia)^3^.

The cause of OLP is unknown and includes specific (cytotoxic action of TCD8+ lymphocytes) and non-specific (activation of metalloproteinases) inflammatory mechanisms. Both mechanisms culminate in the accumulation of T lymphocytes in the lamina propria, basement membrane injury, intraepithelial migration of T cells, and keratinocyte apoptosis^4^.

Classically, the clinical presentations of OLP include papular, reticular, annular, atrophic, erythematous, bullous, and erosive lesions, which reflect variations in the intensity and duration of the inflammatory process^3^. The same patient may present with more than one clinical form of the disease, and the predominant morphology may change over time^3^. The sites most often affected by OLP are the buccal mucosa, gingiva, back of the tongue, labial mucosa and lip vermilion. The erosive forms are the most clinically relevant, as they cause great discomfort and pain.

The present study describes the cases and the clinical-pathological and therapeutic experience with OLP treated at the Stomatology Outpatient Clinic of the Division of Dermatology of Hospital das Clínicas, Faculty of Medicine, Universidade de São Paulo. When present, the simultaneous involvement of other cutaneous areas is also reported and studied.

## Method

A retrospective, longitudinal and descriptive study that included all patients diagnosed with oral lichen planus evaluated at the Stomatology Outpatient Clinic of the Dermatology Division of Hospital das Clínicas, Faculty of Medicine, Universidade de São Paulo was carried out. The medical records of 201 patients seen from 2003 to 2021 were evaluated and cases with clinical and/or histopathological diagnosis of OLP were included. Cases of lichenoid lesions similar to OLP, such as lichenoid graft-versus-host disease, lichenoid mucositis due to dental amalgam, lichenoid drug eruptions, and paraneoplastic pemphigus with a lichenoid pattern were not included in the study. The retrospective review of the medical records of confirmed cases was carried out to obtain the following variables: age at diagnosis, gender, morphotopographic features, clinical picture, performed treatments and presence of associated oral neoplasia.

## Results

The main findings are shown in Table 1.

**Table 1:** Main characteristics of the studied cases of oral lichen planus.

**Subjects, n**	201
Studied period (years)	18
**Characteristics**	
Sex	
Male	31.4% (63)
Female	68.3% (138)
Mean age, ±standard deviation	55 years (9‒86 years), ±14.0225
0‒19 years	2
20‒39	25
40‒59	96
60‒79	72
≥80	6
**Location of lichen planus**	
Oral only	59.7% (120)
Plus skin	23.3% (47)
Plus genital area	4.9% (10)
Plus nails	2.5% (5)
Plus scalp	1% (2)
2 or more locations	7% (14)
**Location of oral lesions**	
Buccal mucosa	67.6% (136)
Tongue	41.8% (84)
Gingiva	32.3% (65)
Lower lip	7.5% (15)
Upper lip	1.5% (3)
Palate	1% (2)
**Characteristics of active lesions (with different lesions being common in the same patient)**	
Leukokeratotic (papular, arboriform, annular)	68.1% (137)
Erosive	33.3% (67)
Desquamative gingivitis	20.4% (41)
**Treatment**	
Topical	
Corticosteroids	60.7% (122)
Calcineurin inhibitors	6.5% (13)
Oral lubricants (sequelae)	17.4% (35)
**Systemic (see text)**	
Prednisone	12.9% (26)
Thalidomide	4% (8)
Azathioprine	1.5% (3)
Mycophenolate-mofetil	1% (2)
Methotrexate	0.5% (1)

The diagnosis was confirmed by histopathology in 77.1% of cases and a biopsy was performed in oral lesions in 64% of patients. In the others, the procedure was performed in LP lesions on other topographies, and the correlation was made with the oral disease. In 22.9% of the cases, the diagnosis was confirmed only through clinical examination, due to abandonment before biopsy, or, more frequently, because the biopsy had already been performed in another service. Direct immunofluorescence examination was performed in all oral mucosa biopsies done by the authors and showed positivity for IgM in cytoid bodies located in the lamina propria in all cases.

Treatment was performed in 82.5% of cases, guided by disease severity and symptomatology. Of 166 treated patients, exclusive topical treatment, either with corticosteroids (122 cases) or calcineurin inhibitors (13), was sufficient to control 80.7% of the cases (some patients received more than one drug). The remaining 17.4% of the patients treated topically comprised cases with long-term evolution, without active lesions, in whom only atrophic sequelae and lingual depapillation were observed, for which only mouthwashes with 10% urea in water were used for symptom relief.

Systemic treatment was instituted in 19.3% of the cases, and it was indicated solely due to the OLP condition in only 3.6% of the patients (cases where the involvement extended to almost the entire mucosa, making it impossible to apply the drug locally). In the other cases, the indication for systemic medication was due to severe simultaneous involvement of other sites (skin, genital mucosa, nails). Of the systemic drugs, the most frequently prescribed was prednisone (26 patients) followed by thalidomide (eight), azathioprine (three), mycophenolate mofetil (two) and methotrexate (one). Some of these patients used topical treatment simultaneously.

Squamous cell carcinoma associated with OLP lesions was observed in eight patients (4%), with histopathological confirmation. Among these, ages ranged between 55 and 73 years, with five female patients and three male patients. In all of them, OLP showed a long evolution with cicatricial sequelae in the mucosa.

## Discussion

There are very few reports of OLP series in Brazil. Most publications on the disease come from the dentistry literature^2^, which unfortunately does not address the disease regarding all of its aspects and fails to report on extra-oral manifestations. A recent Brazilian series reported 41 patients, with no mention of affected sites other than the oral mucosa^5^. The largest international series published by dermatologists also did not highlight extracutaneous involvement^6^.

Extraoral involvement was concomitantly observed in 40% of the patients in the present series, a high number, which may be due to the fact that the data comes from a high-complexity dermatology service. Almost all of these cases had severe mucosal involvement and were referred for treatment by the Stomatology group during the follow-up of LP in other cutaneous sites. Some cases can be characterized as the so-called “vulvo-gingival syndrome”, which can develop important sequelae.^3^ Cases with predominantly cutaneous involvement and minor oral lesions end up not being treated by the authors’ group. This aspect may constitute a more serious bias in the present series if the entire universe of OLP cases in the population is considered.

There was a clear predominance of patients over 50 years old, with only two cases under 20 years old. The largest series of pediatric cases of OLP ever published included eight patients; however without specifying the number of cases covering all age groups from which the series was obtained^7^.

**Figure 1:**
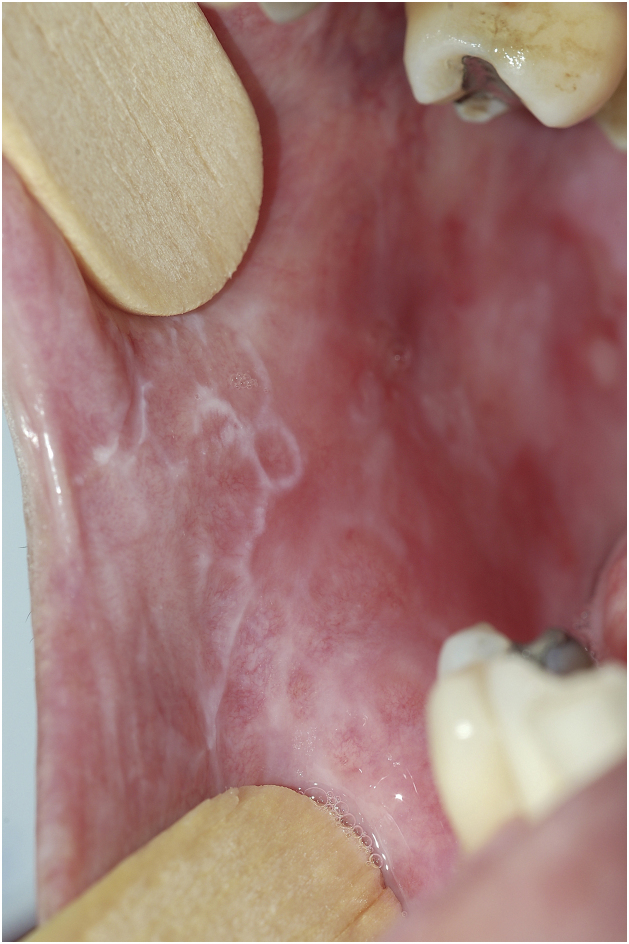
Isolated papular, reticular and annular lesions in the same patient. They can be considered together (“leukokeratotic lesions”) because there is no difference in the approach. There are also erosive and atrophic areas.

It is believed that the traditional classification of OLP lesions into multiple “clinical forms” (papular, reticular, annular, keratotic, erosive, atrophic), used in most publications^2^, ^5^, does not have much influence on case management, since all these presentations simply represent differences regarding intensity, velocity and time of the inflammatory process in the mucosa. It is very common for the patient to present more than one of these patterns simultaneously (Fig. 1). Therefore, the cases included in the present series were stratified by a parameter based on the understanding of the pathological process, into leukokeratotic (stable, little symptomatic), erosive (intense, symptomatic) and atrophic (with sequelae, long-term) presentations, since these three categories will show the main variables in terms of symptomatology, therapeutic indication, and prognosis, thus influencing treatment. Thus, approximately half of the patients had symptomatic presentations leading to sequelae, although many presented papular-keratotic lesions simultaneously (see Table 1).

The so-called desquamative gingivitis is a characteristic erosive presentation, with involvement of the gingiva. It is a difficult clinical diagnosis when the condition affects only this region. This is due to the fact that, in addition to OLP, this aspect can also be observed in pemphigus vulgaris and pemphigoid of the mucous membranes, and histopathology is mandatory for attaining a correct diagnosis^8^.

The most frequently used diagnostic test for OLP is still histopathology, performed in the vast majority of the present cases. The criteria for the histopathological diagnosis of the disease are well established^3^, and the biopsy is easy to perform. Direct immunofluorescence uniformly depicts positivity for IgM in cytoid bodies located in the lamina propria; however, this test can be perfectly dispensed with in the diagnosis of OLP. In recent years, non-invasive methods such as confocal reflective microscopy have been attempted in the diagnosis of mucosal inflammatory diseases, with very promising results^8^.

The authors did not systematically investigate the presence of hepatitis C virus infection in their patients^6^. In their experience, it is unusual to detect this virus infection in OLP cases; more commonly a few patients, known to be carriers of the virus, may develop OLP during the course of the disease, thus not justifying this investigation in all cases.

Presentations considered severe (erosive LP and desquamative gingivitis) were observed in 54% of the patients, were treated more incisively and closely followed. These patients very often, during treatment and after control of the disease, developed mucosal atrophic-cicatricial sequelae; once disease activity ceased, they were only treated symptomatically and submitted to long-term follow-up, aiming to attain an early detection of complications (see below).

A recent review on OLP proposes an algorithm for OLP treatment, with measures ranging from simple observation, going through topical therapy with corticosteroids or calcineurin inhibitors, and reaching systemic treatments, starting with oral corticosteroid therapy, and progressing to immunosuppressants and biological agents^9^. In the authors experience and present series, the use of potent topical corticosteroids (clobetasol) in an orabase vehicle was sufficient to control almost all cases of symptomatic OLP. Systemic treatment was necessary only in cases in which the lesions occupied multiple mucosal sites, making it impossible to apply the ointment, and in cases with significant extraoral manifestations. The good OLP response to topical medication is due to the fact that the lesions requiring treatment are erosive ones (very symptomatic), and in these, the inflammatory infiltrate is easily reached by the medication, due to the loss of the epithelial lining of the mucosa. From a practical point of view, the medication is applied three times a day and, at night, a nystatin solution is applied to prevent the appearance of oral candidiasis, which is very common when using potent topical corticosteroids on the mucosa.

**Figure 2:**
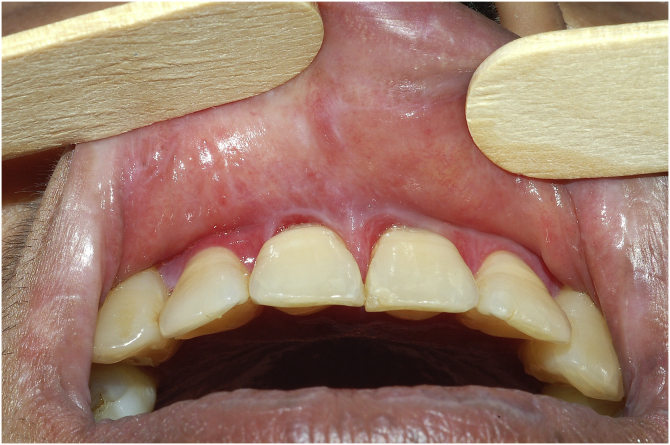
Synechiae in the gingival sulcus, associated with erosions, due to lichen planus of long evolution.

Long-term follow-up of patients with OLP is necessary due to disease chronicity. Even more than flares of disease activity, what is observed over the years is the appearance of sequelae, characterized by mucosal atrophy, tongue depapillation, leukokeratotic scars, and even synechiae affecting the gingival sulcus or the lingual frenulum (Fig. 2). These sequelae are sometimes very uncomfortable for the patient, especially tongue depapillation, which results in loss of protection and superficialization of taste receptors, leading to intense local sensitivity to food and liquids. Dental hygiene difficulties may occur due to synechiae in the gingival sulcus.

These cicatricial areas, in the long term, may become the site of complications, such as the onset of squamous cell carcinoma (SCC). Many publications have discussed the so-called “malignant potential” of OLP. Several studies try to associate an alleged increased risk of “malignant transformation” of OLP lesions into SCC, with this “risk” varying between 0.4% and 5%, with observation periods ranging from six months to 20 years^10^. In fact, what rarely occurs is a phenomenon that has long been described and recognized by dermatologists: the eventual onset of SCC in areas of vicious healing and sequelae of different dermatoses. Classic examples are the appearance of SCC on cicatricial sequelae of burns, lupus vulgaris, discoid lupus erythematosus, dystrophic epidermolysis bullosa, hidradenitis and porokeratosis, among other diseases, with this tumor being called “Marjolin's ulcer”^11^. SCC will only appear in cases of long-term OLP and the presence of intense cicatricial sequelae in the mucosa; hence, the importance of long-term follow-up. SCC is very rare in association with cutaneous LP, as the latter rarely develops cicatricial sequelae, but it can occasionally occur^12^.

In the present series, the finding of eight cases of SCC associated with OLP, a relatively high number, may be due to the many years of follow-up of cases that had atrophic sequelae. Of these, seven cases had small, easily treatable lesions. Only one female patient had an extensive vegetating tumor, which had already been observed in her first appointment.

## Conclusion

This is the largest Brazilian series of OLP from a dermatology service. Of the significant data obtained, the authors emphasize the strong predominance in women over 50 years of age, the excellent response to local corticosteroid therapy in the vast majority of cases, and the high prevalence of atrophic sequelae, showing the chronicity and tissue destruction potential of this disease.

## Financial support

None declared.

## Authors' contributions

Aline Erthal: Data collection; writing of the manuscript.

Silvia Vanessa Lourenço: Final review; study planning.

Marcello Menta Simonsen Nico: review of the literature; final review; study planning.

## Conflicts of interest

None declared.
